# miR-216b regulation of c-Jun mediates GADD153/CHOP-dependent apoptosis

**DOI:** 10.1038/ncomms11422

**Published:** 2016-05-13

**Authors:** Zhenhua Xu, Yiwen Bu, Nilesh Chitnis, Costas Koumenis, Serge Y. Fuchs, J. Alan Diehl

**Affiliations:** 1 Department of Biochemistry, Hollings Cancer Center, Medical University of South Carolina, 86 Jonathan Lucas Street, 3400, Charleston, South Carolina 29425, USA; 2 Department of Radiation Oncology, Perelman School of Medicine, University of Pennsylvania, Philadelphia, Pennsylvania 19104, USA; 3 Department of Animal Biology, School of Veterinary Medicine, 380 S. University Avenue, Philadelphia, Pennsylvania 19104, USA

## Abstract

The ability of the unfolded protein response, UPR, to regulate cell homeostasis through both gene expression and protein synthesis has been well documented. One primary pro-apoptotic protein that responds to both PERK and Ire1 signalling is the CHOP/GADD153 transcription factor. Although CHOP deficiency delays onset of cell death, questions remain regarding how CHOP regulates apoptosis. Here, we provide evidence demonstrating that CHOP/GADD153-dependent apoptosis reflects expression of micro-RNA, miR-216b. MiR-216b accumulation requires PERK-dependent induction of CHOP/GADD153, which then directly regulates miR-216b expression. As maximal expression of miR-216b is antagonized by Ire1, miR-216b accumulation reflects the convergence of PERK and Ire1 activities. Functionally, miR-216b directly targets c-Jun, thereby reducing AP-1-dependent transcription and sensitizing cells to ER stress-dependent apoptosis. These results provide direct insight into the molecular mechanisms of CHOP/GADD153-dependent cell death.

## Abstract (web summary)

The transcription factor CHOP/GADD153 regulates apoptosis in response to the unfolded protein response. Here the authors show that CHOP/GADD153 regulates the expression of miR-216b, which targets c-Jun and sensitizes cells to ER stress-dependent apoptosis.

## Graphical abstract

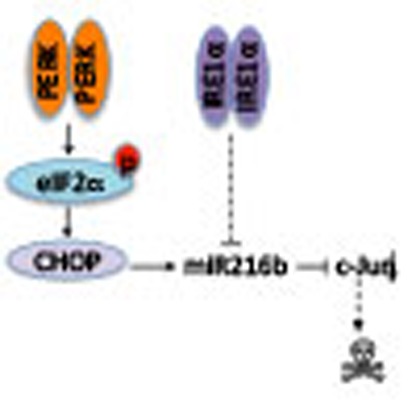

The endoplasmic reticulum (ER) serves as a nexus for the folding and maturation of proteins that transit the secretory pathway. Proteins are imported co-translationally whereupon they undergo additional energy intensive post-translational modifications before folding and maturation. Protein folding is both regulated and sensed by ER resident chaperones, such as Grp78/BiP and Grp94 (refs 1, 2, 3, 4, 5). Under conditions where protein production out paces protein folding capacity, for example, under nutrient-deficient conditions as might occur during neoplastic growth, misfolded proteins accumulate in the ER triggering a stress sensing/adaptive pathway referred to as the unfolded protein response (UPR).

Mammalian cells contain three ER transmembrane proteins that function as proximal effectors of the UPR. Inositol-requiring enzyme, Ire1, isoforms (α, ubiquitously expressed; β tissue restricted) are composed of a luminal domain that senses stress, a single transmembrane domain, and a cytosolic tail that contains both a protein kinase domain and an RNase domain^6^,^7^. Ire1 triggers increased expression of numerous ER chaperones through activation of the X-box-binding protein 1 (Xbp1) transcription factor^8^,^9^. Accumulation of Xbp1 is mediated by the RNase and splicing function of Ire1; stress-induced splicing generates a shorter Xbp1 mRNA that is more efficiently translated^10^,^11^. Protein kinase RNA-like ER kinase (PERK) is activated in a manner analogous to the Ire1; it catalyses serine 51 phosphorylation of eIF2α resulting in repression of protein synthesis^12^,^13^,^14^. The third signalling component are the transmembrane transcription factors ATF6α/β (refs 15, 16). Although normally tethered to the ER, upon stress, ATF6 migrates to the trans-Golgi, where it is processed by S1P and S2P proteases to release the N-terminal DNA-binding transcription factor domain^17^,^18^,^19^. Activation of PERK, Ire1 and ATF6 is mediated by sequences within their respective luminal domains to which the ER chaperone, BiP, binds^20^. Activation is triggered by increased unfolded proteins, which compete for BiP binding.

The ability of the UPR to regulate protein synthesis, gene expression and contribute to cell homeostasis has been well documented. However, an added layer of communication that entails the regulation of small non-coding RNAs, micro-RNAs, was recently revealed. Micro-RNAs are 20–22 nucleotide molecules that generally impact protein expression by virtue of their capacity to degrade target mRNAs or reduce their efficiency of translation^21^,^22^. During the past 4–5 years, work from several groups has revealed that all three branches of the UPR regulate specific subsets of micro-RNAs^23^,^24^,^25^,^26^,^27^. The modes of regulation include Ire1-mediated micro-RNA degradation^25^, ATF4-dependent and ATF6-dependent transcription^27^. The consequences of micro-RNA induction include the modulation of protein expression as would be expected, while certain micro-RNAs also directly contribute to regulated gene expression^27^.

The UPR can communicate both pro-adaptive and pro-apoptotic signals. Ambiguities remain regarding whether an adaptive or apoptotic signal is generated given that all three transducers have been associated with both survival and apoptosis under varying conditions (9,28,29,30,31,32,33,34). One primary pro-apoptotic protein that responds to both PERK and Ire1 signalling is the CHOP/GADD153 transcription factor. Although CHOP deficiency delays onset of cell death, questions remain regarding how CHOP regulates apoptosis. The data presented here reveal evidence for CHOP-dependent micro-RNA, miR-216b, which subsequently suppresses c-Jun expression thereby, triggering cell death.

## Results

### MiR-216b is induced by the UPR

**Figure 1:**
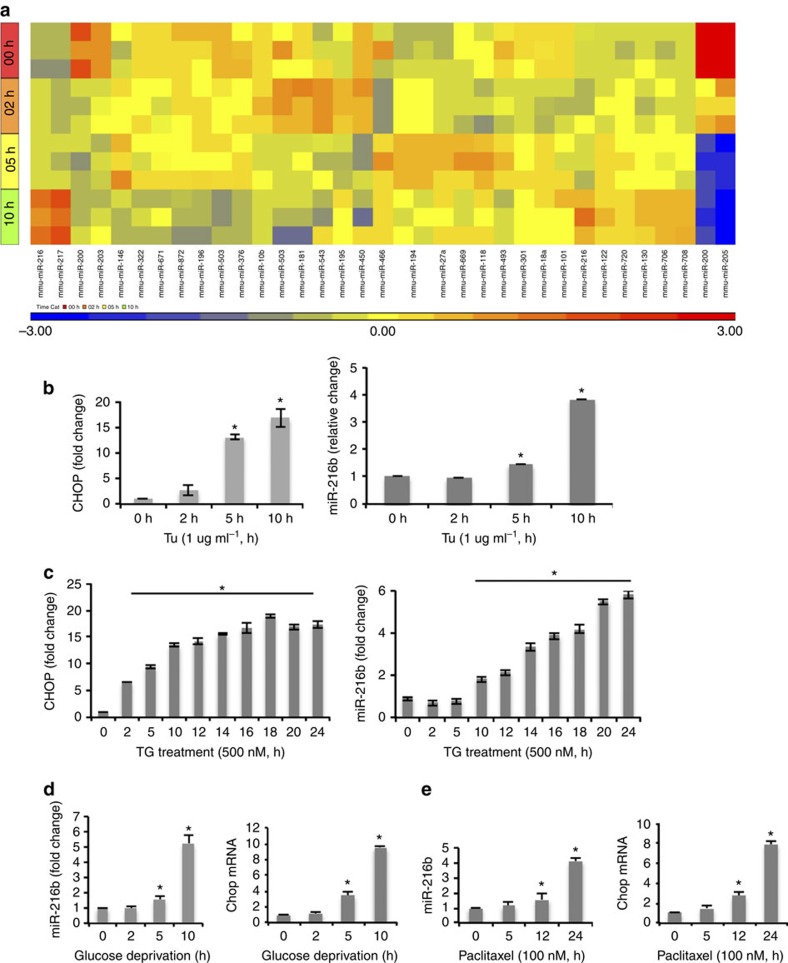
ER stress induced miR-216b expression. (**a**) Heat map depicting differentially expressed microRNAs (at least two-fold) following exposure to tunicamycin (Tm) as indicated. (**b**,**c**) qPCR analysis of CHOP mRNA levels and miR-216b expression in NIH3T3 cells treated with Tm (1 μg ml^−1^) or thapsigargin (TG, 500 nM) for indicated times. Averages are calculated from three independent experiments. (**d**) qPCR analysis of miR-216b expression and CHOP mRNA levels in NIH3T3 cells cultured in low-glucose medium for indicated times. (**e**) qPCR analysis of miR-216b expression and CHOP mRNA levels in U2OS cells treated with paclitaxel (100 nM) for indicated times. Data are mean±s.d. of three independent experiments. Statistical analysis was analysed by Student's *t*-test. (*P<0.05, treatment versus non-treatment).

To identify micro-RNAs that are induced following prolonged ER stress, RNA harvested from NIH3T3 cells challenged with tunicamycin (Tu) was subjected to micro-array analysis. MiR-211, not represented in this figure, induction was assessed as an internal control. MiR-216b and miR-217 exhibited increased expression throughout the time course (Fig. 1a). Several groups have evaluated miR-216b expression and potential function in various cancers including colorectal cancer, hepatocellular carcinoma and nasopharyngeal carcinoma^35^,^36^,^37^; we therefore chose to assess miR-216b regulation and function during ER stress. To verify the microarray data, RNA was collected from Tm-challenged NIH3T3 cells, assessed for CHOP induction as a control for UPR activation and for miR-216b. CHOP induction was noted by 2 h and consistent with our microarray data, we observed an accumulation of miR-216b (Fig. 1b). Accumulation of miR-216b was evident following the exposure of cells to an independent inducer of ER stress, thapsigargin (TG; Fig. 1c). MiR-216b expression was also induced in response to chemotherapeutic agents that generate ER stress^38^ and physiologically relevant low glucose (Fig. 1d,e).

### PERK-dependent induction of miR-216b

**Figure 2:**
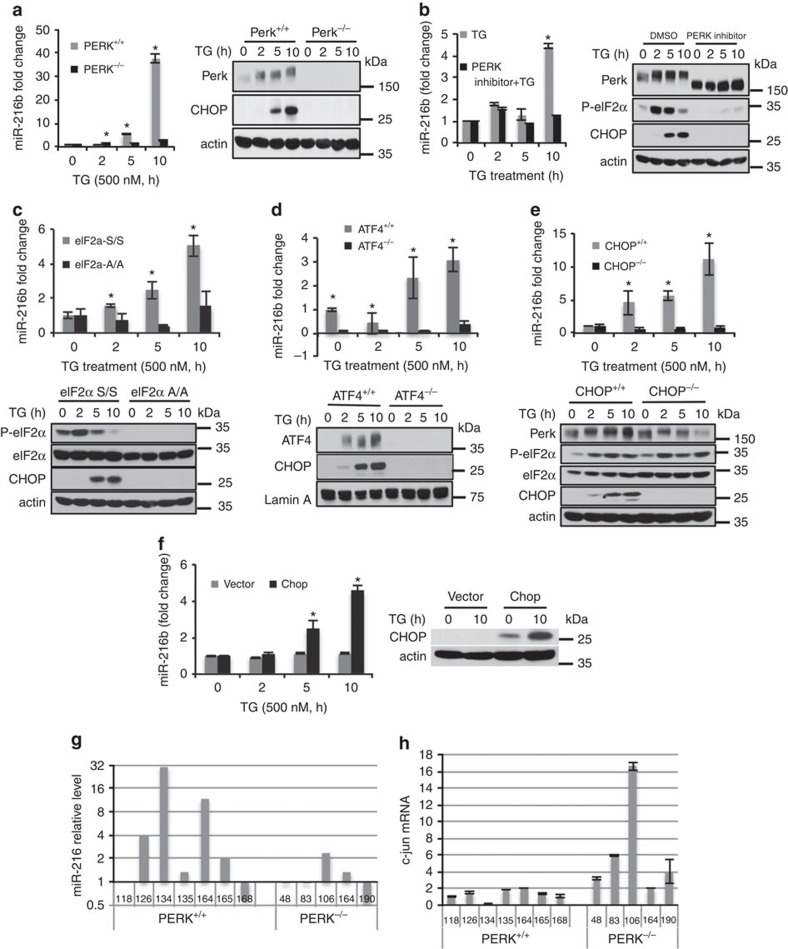
PERK-dependent miR-216b induction. (**a**) PERK^+/+^ and PERK^−/−^ MEFs were treated with 500 nM TG for indicated times. MiR-216b was assessed by qPCR (left graph); PERK and CHOP were assessed by immunoblot (right). (**b**) MiR-216b levels were quantified by qPCR following exposure of cells to thapsigargin and a small-molecule PERK inhibitor (left). PERK, eIF2α-p and CHOP induction were assessed by immunoblot (right). (**c**–**e**) MEFs of the indicated genotype were treated with TG (500 nM) for indicated intervals. Protein extracts from these cells were immunoblotted for the proteins as indicated (lower panels) and miR-216b levels were quantified by qPCR (upper panels; *n*=3). (**f**) CHOP^−/−^ MEFs were transfected with vector or CHOP and 2 days later treated with TG (500 nM) for indicated intervals. Protein extracts from these cells were immunoblotted for CHOP and miR-216b levels quantified by qPCR (*n*=3). (**g**) MiR-216b expression and (**h**) c-Jun mRNA levels were analysed in MMTV-Neu tumours from either PERK^+/+^ or a PERK^−/−^ background. Data represent mean±s.d. of three independent observations. Statistical significance was analysed analysed by Student's *t*-test. (*P<0.05, WT versus −/−).

Because PERK was previously implicated as a regulator of micro-RNA accumulation during ER stress, we assessed its contribution to the regulation of miR-216b expression. Wild-type or PERK deficient murine embryonic fibroblasts^39^ were treated with TG (500 nM), and expression of miR-216b was assessed by quantitative PCR (qPCR). PERK deficiency was confirmed by western blot analysis using a PERK-specific antiserum and through assessment of CHOP accumulation following stress (Fig. 2a). MiR-216b accumulation was noted in wild type but not PERK^−/−^ cells (Fig. 2a). To corroborate the dependence of miR-216b expression on PERK activity, we used GSK2606414 (GSK414), a highly specific PERK small-molecule inhibitor^40^,^41^. GSK414 effectively inhibited PERK activation and miR-216b induction (Fig. 2b).

PERK-dependent regulation of gene expression is often dependent upon increased accumulation of the ATF4 transcription factor, whose accumulation is in turn dependent upon eIF2α phosphorylation^13^,^14^,^42^,^43^,^44^. To address the contribution of eIF2α and ATF4, wild-type mouse embryonic fibroblasts (MEFs) or MEFs isolated from eIF2αS51A knockin embryos^45^ or from ATF4^−/−^ embryos^46^ were challenged with TG. MiR-216b induction was absent in both eIF2αS51A and ATF4^−/−^ backgrounds but was induced in wild-type MEFs (Fig. 2c,d) demonstrating a role for ATF4 in the regulation of miR-216b expression.

As ATF4 accumulates within the first 2–4 h of ER stress^46^,^47^, and miR-216b accumulation occurs primarily between 5 and 10 h, we considered the possibility that ATF4-dependent regulation was indirect. We focused our attention on CHOP, a direct transcriptional target of ATF4 (ref. 43). MiR-216b induction was abolished in *chop* knockout MEFs (Fig. 2e). Let7a accumulation was assessed as a nonspecific control (Supplementary Fig. 1). Consistent with CHOP regulation, restoring CHOP expression in *chop*^−/−^ MEFs rescued stress-dependent miR-216b expression (Fig. 2f). These data demonstrate that ER stress-dependent induction of miR-216b reflects a pathway minimally composed of PERK, eIF2α, ATF4 and CHOP.

We also assessed miR-216b expression in a panel of MMTV-Neu tumours that were derived in mice with either wild-type PERK or from mice in which PERK was excised from mammary epithelium with Cre recombinase^23^,^48^. In this cohort, miR-216b expression was significantly reduced in PERK-deficient tumours and this correlated with c-Jun expression levels, providing evidence for PERK-dependent induction of miR-216b *in vivo* (Fig. 2g,h).

### MiR-216b is a direct CHOP transcriptional target

**Figure 3:**
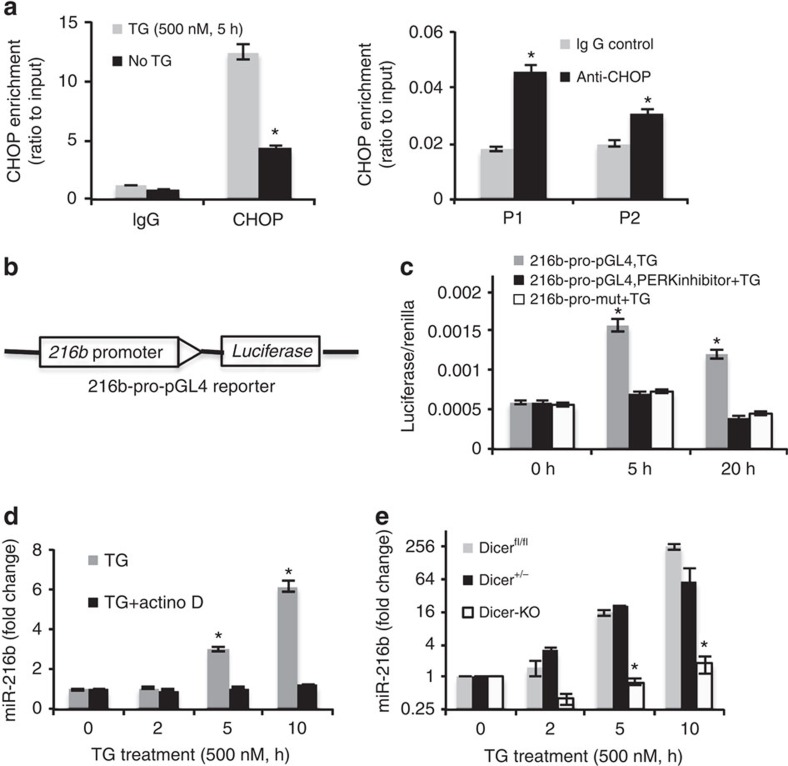
CHOP binds to miR-216b promoter region. (**a**) Occupancy of CHOP on the promoter of mouse miR-216b. NIH3T3 cells (left panel) and U2OS (right panel) were treated with/without TG for 5 h, followed by ChIP assay using IgG or an anti-CHOP antibody. Precipitated DNA was subjected to qPCR analysis. All ChIP data represent the mean and standard error of the mean from three independent experiments. (**b**) Schematic representation of miR-216b promoter reporter (216b-pro-pGL4; left panel). (**c**) 293T cells were co-transfected with 216b-pro-pGL4, 216b-mut-pGL4 and Renilla-Luc as an internal control. 48 h post transfection, cells were treated with PERK inhibitor followed by TG treatment for intervals as indicated. Values are the ratios of firefly to Renilla luminescence (mean±s.d. of triplicate experiments). (**d**) NIH3T3 cells were treated with/without actinomycin D (2 μg ml^−1^) for 1 h and challenged with 500 nM TG as indicated. MiR-216b levels were measured with qPCR (mean±s.d., *n*=3). (**e**) Dicer^flox/flox^, Dicer^+/−^ and Dicer^−/−^ MEFs were treated with 500 nM TG, qPCR assessment of miR-216b (left) and chop mRNA (right). Data represent mean±s.d. of three independent experiments and statistical analysis was analysed by Student's *t*-test (*P<0.05).

Interestingly, ATF4 and CHOP share a set of target genes, with the preference of binding to the similar motifs (GCATCAT/G)^47^. Using an unbiased motif search of ∼2 kbp upstream of mouse miR-216b, we found a potential binding site for ATF4 and/or CHOP at −266 to −259 (Supplementary Fig. 2a). To evaluate potential binding to this motif, we performed chromatin immunoprecipitation (ChIP). ChIP with either anti-CHOP (Fig. 3a, right and left panel) or anti-ATF4 (Supplementary Fig. 2b) followed by qPCR revealed that TG treatment triggered a significant enrichment of CHOP on the miR-216b promoter relative to unstressed cells and control IgG. Under the same conditions, there was no apparent enrichment of ATF4 on the miR-216b promoter, although binding of ATF4 to the CHOP promoter was confirmed (Supplementary Fig. 2b,c).

To further evaluate whether the upstream region from −266 to −259 region is responsible for miR-216b regulation, we used a reporter assay system where 1 kbp of upstream miR-216b sequence was cloned upstream of the firefly luciferase gene (Fig. 3b). Following expression in 293T cells, luciferase activity was induced following TG treatment (Fig. 3c). Luciferase activity was abolished by GSK414 (Fig. 3c, black bars) demonstrating PERK dependence. Alteration of the CHOP-binding site through site-directed mutagenesis abrogated UPR-dependent induction (Fig. 3c-white bars). Actinomycin D (2 μg ml^−1^) treatment before exposure to TG treatment suppressed miR-216b expression, consistent with transcription dependence of miR-216b induction (Fig. 3d). To determine whether miR-216b biogenesis is Dicer dependent we utilized inducible Dicer Knockout (Dicer^fl/fl^) or heterozygous (Dicer^+/fl^) MEFs after deletion of the floxed allele(s) with hydroxytamoxifen^49^. After treatment with TG, miR-216b expression was significantly lower in Dicer-KO cells than that in Dicer^fl/fl^ or Dicer^+/−^ cells demonstrating Dicer dependence (Fig. 3e).

### Ire1α inhibits miR-216b expression

**Figure 4:**
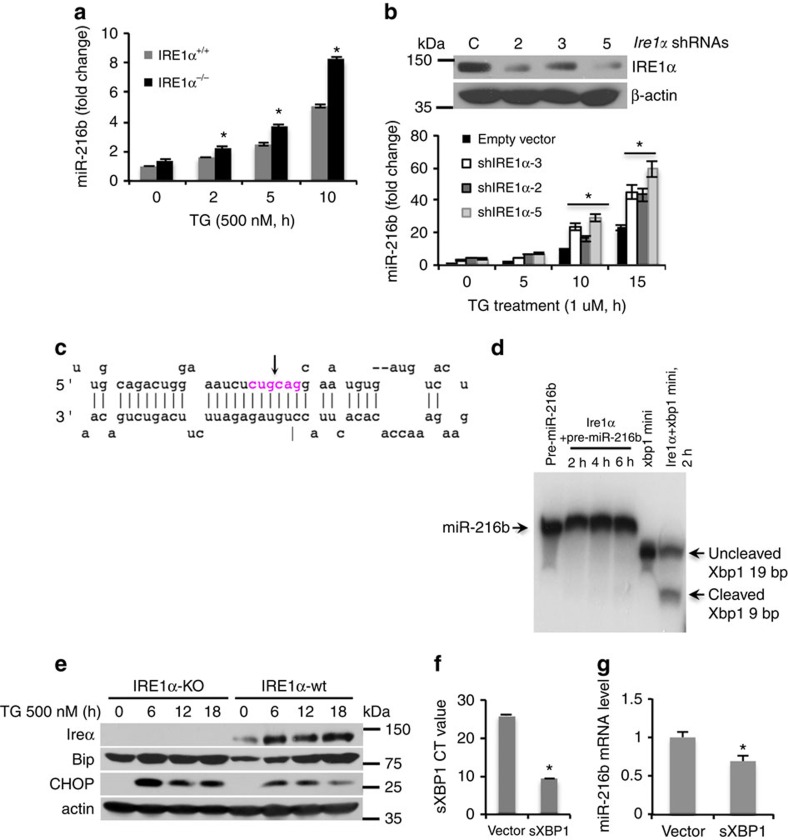
Ire1α suppresses miR-216b expression. (**a**) Ire1α^−/−^ and Ire1wt MEFs were treated with 500 nM TG and miR-216b (right) was measured by qPCR. *N*=3. (**b**) Ire1α was knocked down using three independent shRNAs 2, 3, 5 followed by treatment of cells with 500 nM TG. Ire1α knockdown was confirmed by immunoblot, and miR-216b levels were quantified by qPCR. (**c**) Illustration of the potential Ire1α cleavage sites within pre-miR-216b. (**d**) γ-^32^PATP-labelled pre-miR-216b or Xbp-1 oligonucleotides were incubated with recombinant protein Ire1α (aa 465–977) for 2, 4 and 6 h, and cleavage products were resolved by a urea-polyacrylamide gel electrophoresis and visualized by autoradiography. (**e**) Ire1α^−/−^ and Ire1wt MEFs were treated with TG (500 nM) for indicated intervals. Ire1α, Bip and CHOP were assessed by immunoblot. (**f**) Xbp1s expression and (**g**) miR-216b levels in U2OS cells transfected with vector or Xbp1s were quantified by qPCR following exposure of cells to thapsigargin. All panels provide values that are the average of three independent experiments and error bars indicate standard deviation among all the replicates. Statistical analysis was analysed by Student's *t*-test (*P<0.05).

Mammalian Ire1α initiates downstream signalling through unconventional splicing of the transcription factor *Xbp1* (ref. 10), phosphorylation of downstream targets or via regulated Ire1-dependent decay (RIDD) of multiple substrates^50^. Ire1α RIDD activity also contributes to the regulation of micro-RNA accumulation during ER stress^25^. To determine the contribution of Ire1α in miR-216b expression, we treated Ire1α knockout MEFs (Ire1α^−/−^) and wild-type MEFs with TG. A significant increase in miR-216b induction in Ire1α knockout cells relative to wild type was observed (Fig. 4a). As an independent analysis, we used three different short hairpin RNAs (shRNAs) targeting Ire1α; notably, all three shRNAs reduced Ire1α expression thereby permitting increased accumulation of miR-216b following ER stress (Fig. 4b).

As the miR-216b stem-loop contains a putative Ire1α cleavage site (CUGCAG (ref. 51); Fig. 4c), we considered whether miR-216b might be a RIDD substrate for Ire1α. Recombinant Ire1α (aa 465–977), did not cleave a labelled miR-216b precursor (incubation up to 6 h), but did cleave Xbp1 (Xbp1-mini, 5′-CUGAGUCCGCAGCACUCAG-3′; Fig. 4d). As an independent approach, we transfected HA-tagged Ire1α wt, Ire1α-509A and Ire1α-907A into 293T cells followed by immunoprecipitation with an anti-HA antibody. Immune-purified Ire1α wt cleaved the Xbp1 single stem-loop mini-substrate, whereas Ire1α-509A and IRE1α-907A were inactive as expected (Supplementary Fig. 3). No specific cleavage of miR-216b was noted suggesting that Ire1α-dependent suppression of miR-216b is indirect. Published work has demonstrated that CHOP expression is elevated in Ire1^−/−^ cells^52^, providing a possible explanation for increased miR-216b expression. Indeed, CHOP expression was more abundant in Ire1^−/−^ cells (Fig. 4e) correlating with increased miR-216b levels. Finally, to address whether Xbp1 suppress miR-216b accumulation, we enforced expression of spliced Xbp1 expression in Ire1^−/−^ cells (Fig. 4f). Enforced sXbp1 resulted in reduced miR-216b consistent with sXbp1-suppressing miR-216b (Fig. 4g).

### MiR-216b regulates c-Jun accumulation during ER stress

**Figure 5:**
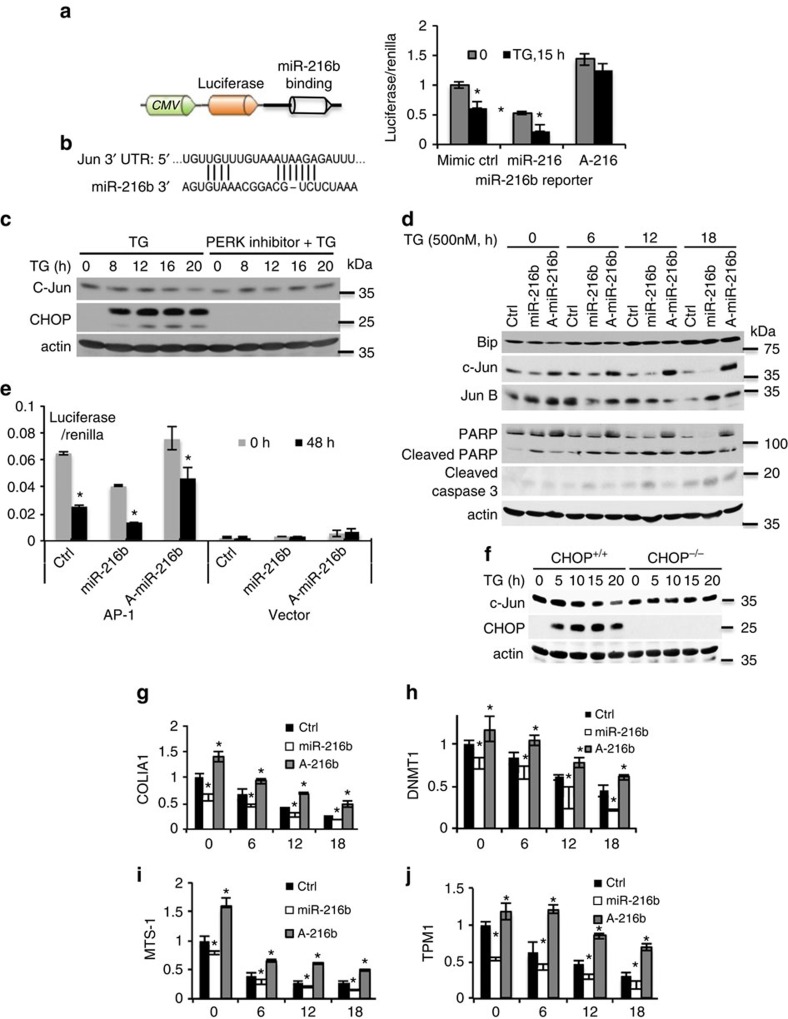
MiR-216b targets the c-Jun 3′UTR. (**a**) miR-216b reporter construct (left) was expressed in mimic control (ctrl), miR-216b or A-miR-216b-expressing U2OS cells. Luciferase activity was measured and normalized to internal transfection control Renilla. Error bars represent standard deviation for three independent experiments. (**b**) MiR-216b seed sequence matches in the c-Jun 3′UTR. (**c**) NIH3T3 cells were treated with PERK inhibitor for 1 h and followed by 500 nM thapsigargin (TG); c-Jun and CHOP protein levels assessed by immunoblot. (**d**) U2OS stable cell lines expressing control, miR-216b or A-miR-216b were treated with 500 nM TG as indicated. Levels of the indicated proteins were measured by immunoblot. (**e**) An AP-1 luciferase reporter was transiently introduced into NIH3T3 cells along with a Renilla control. Cells were co-transfected as indicated with control, miR-216b or A-miR-216b. 48 h post transfection, cells were treated with TG before quantifying luciferase relative to Renilla control. (**f**) CHOP^+/+^ and CHOP^−/−^ MEFs were treated with 500 nM TG for indicated intervals. CHOP and c-Jun were immunoblotted. (**g**–**j**) qPCR analysis of CHOP mRNA levels downstream target genes of c-Jun in U2OS treated as in **d**. Data present the mean of three independent experiments, error bars indicate the standard deviation. Statistical significance was analysed analysed by Student's *t*-test (*P<0.05).

Before searching for targets of miR-216b, a luciferase reporter gene containing three tandem miR-216b-binding seeds in its 3′ untranslated region (UTR) was used to determine whether miR-216b induction was of functional consequence. Treatment of cells expressing the reporter with TG resulted in a 50% reduction in luciferase activity (Fig. 5a; left two columns). Co-transfection with miR-216b mimic also resulted in an ∼50% decrease in reporter gene expression; inclusion of TG reduced expression further by ∼80% (Fig. 5a; middle columns). Luciferase activity increased 1.5-fold when co-transfected with an antagomir, A-miR-216b (Fig. 5a; right columns).

To identify miR-216b targets, a computational algorithm was utilized to search 3′UTR regions of annotated cDNAs for miR-216b seed sequence matches; TargetScan (http://www.targetscan.org), revealed c-Jun as a high relevance target (Fig. 5b). TG exposure resulted in a time-dependent decrease in c-Jun levels (Fig. 5c; quantification Supplementary Fig. 4a). In contrast pre-incubation with a PERK inhibitor ablated c-Jun regulation (Fig. 5c). To assess the contribution of miR-216b to stress-dependent inhibition of c-Jun, U20S cell lines that stably overexpressed an shRNA vector encoding a miR-216b mimic or an anti-miR-216b were generated and subsequently exposed to a time-course of TG treatment. Cells expressing miR-216b mimic exhibited reduced basal c-Jun levels and increased kinetics of c-Jun loss relative to control (Fig. 5d; quantification cJun Supplementary Fig. 4c, cleaved caspase Supplementary Fig. 4b). Conversely, expression of A-miR-216b resulted elevated basal expression of c-Jun and a significant maintenance of c-Jun levels during ER stress (Fig. 5d).

In combination with c-Fos, c-Jun forms the AP-1 early response transcription factor^53^,^54^. It is expected that miR-216b induction should trigger decreased AP-1 activity. To test this hypothesis, a reporter construct wherein luciferase expression is driven by a minimal promoter and 3X AP-1-binding sites (TGAACTCA) was utilized. AP-1-dependent activity was reduced by ER stress (Fig. 5e). The inhibition of AP-1-dependent reporter gene expression was amplified by co-expression of ectopic miR-216b and attenuated by A-miR-216b (Fig. 5e). We also reasoned that if miR-216b induction is CHOP dependent, CHOP deficiency should ablate c-Jun inhibition during ER stress. Consistently, CHOP^−/−^ exposed to ER stress failed to suppress c-Jun after TG treatment (Fig. 5f). Finally, assessment of expression of c-Jun target genes revealed that ER stress and miR-216b reduced expression, whereas A-miR-216b rescued expression (Fig. 5g–j; quantification of cJun Supplementary Fig. 4d). These results revealed a direct relationship between miR-216b expression and c-Jun downregulation following ER stress.

### Suppression of c-Jun sensitizes cells to apoptosis

**Figure 6:**
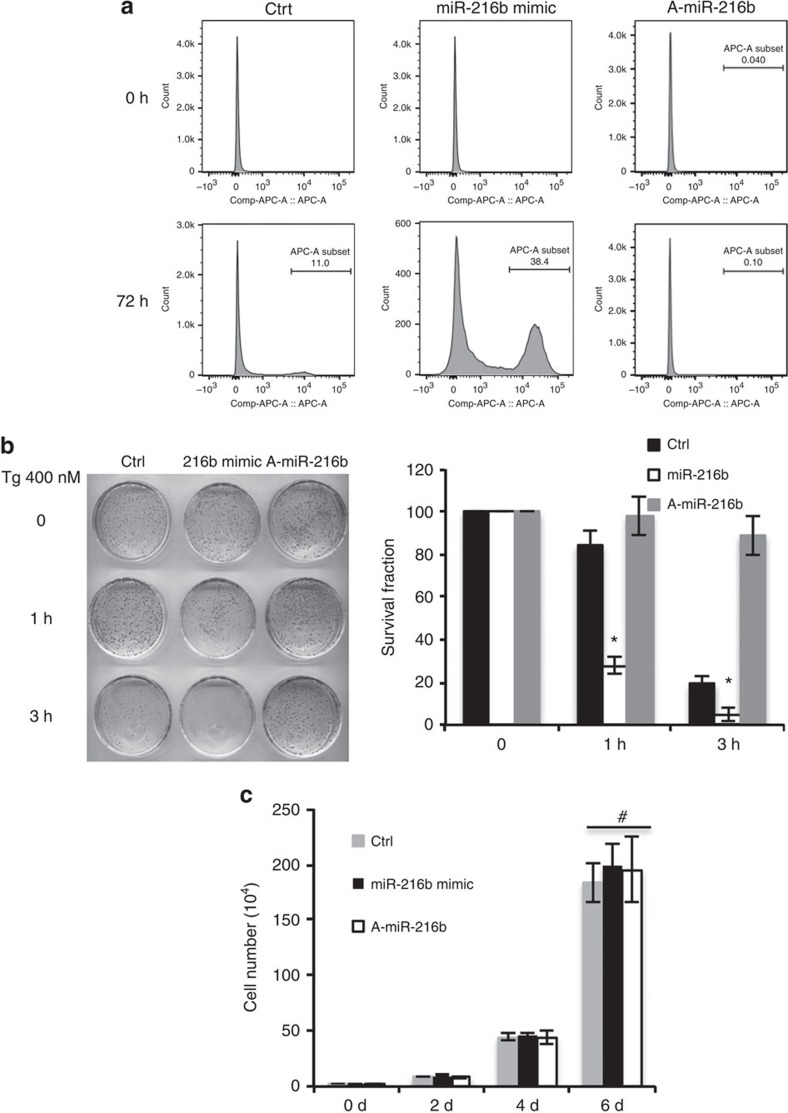
MiR-216b expression sensitizes cells to ER stress. (**a**) Representative FACS analysis histograms of U2OS stable cells expressing control, miR-216b or A-miR-216b after treatment with 500 nm TG. (**b**) U2OS cells stably expressing control, miR-216b or A-miR-216b were plated and treated with 400 nM TG for 1 or 3 h, and then returned to normal growth medium for 12 days (d). Plates were assessed for colony formation by staining with Giemsa staining (left). Quantification of colonies (right). (**c**) Cell doubling was quantified over 6 days. Values are means±s.d. (*n*=3) and statistical significance was analysed by Student's *t*-test. (*P<0.05; ^#^*P*>0.05).

As c-Jun protects cells from ultraviolet and tumour-necrosis factor-α-induced apoptosis through cooperation with NF-κB^55^,^56^, we considered the possibility that miR-216b might regulate ER stress-dependent apoptosis through regulation of c-Jun. Initially, we determined whether modulation of miR-216b altered ER stress-dependent apoptosis. Consistent with miR-216b as a regulator of cell survival, overexpression of miR-216b increased apoptosis induced by TG (Fig. 6a). In contrast, A-miR-216b-expressing cells were largely refractory to the TG-induced apoptosis (Fig. 6a). As an independent measure of cell survival, we exposed control, miR-216b- and A-miR-216b-expressing U2OS to TG for 0 or 3 h following which cells were supplied with fresh medium lacking TG. The cells were allowed to recover for 12 days, at which point surviving cells/colonies were quantified. miR-216b-expressing U2OS cells were significantly more sensitive to stress than control, whereas A-miR-216b-expressing cells were refractory to TG-induced death (Fig. 6b). The effect of miR-216b on recovery from ER stress was also observed with a second ER stressor, Tm (Supplementary Fig. 5). Cell doubling times were not affected by the expression of either A-miR-216b or overexpression of miR-216b mimic confirming that miR-216b is regulating cell viability not cell proliferation (Fig. 6c).

**Figure 7:**
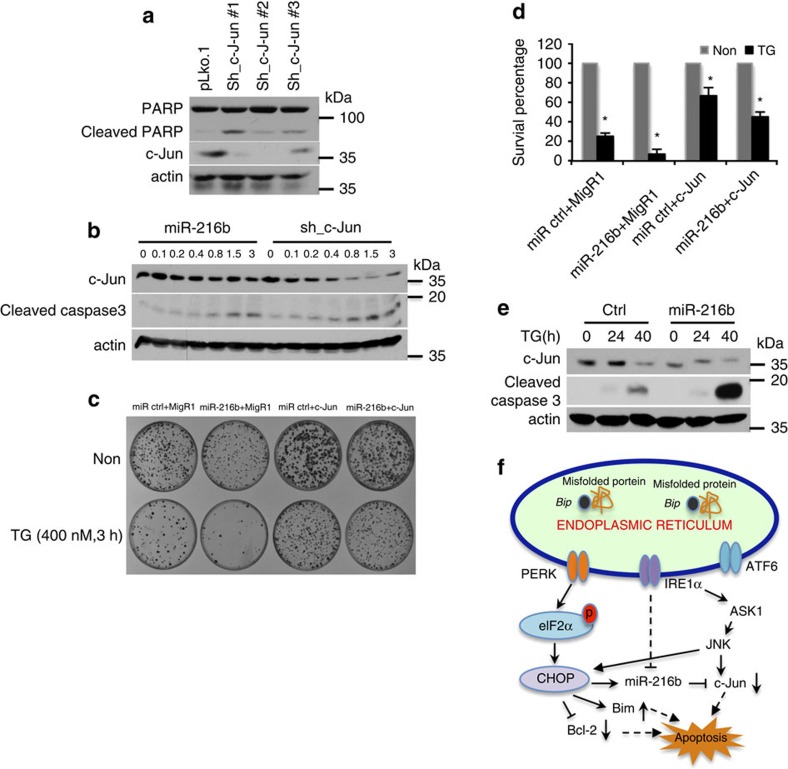
MiR-216b sensitizes cells to ER stress-dependent apoptosis via regulation of cJun. (**a**) c-Jun knockdown with three independent shRNA (top panel). Total and cleaved PARP and cJun were assessed by immunoblot (middle panel). (**b**) U2OS cells were infected with virus encoding either miR-216b or sh-c-Jun # 2 (from panel **a**) at varying viral concentrations (top). C-Jun and cleaved caspase 3 were quantified by immunoblot 48 h post infection. (**c**) U2OS cells stably expressing control, miR-216b or A-miR-216b were infected with retrovirus coding MIGR1 or c-Jun for 3 days, cells were plated at 2 × 10^3^ cells per 60 mm dish, treated with 400 nM thapsigargin (TG) for 1–3 h, and then returned to growth medium for 12 days. (**d**) Quantification of Giemsa-stained colonies from **c**. Average cell survival fractions (TG treatment/untreated). Values are means±s.d. (*n*=3). (**e**) miR-216b was introduced into CHOP^−/−^ cells, treated as indicated with TG and assessed for accumulation of cleaved caspase 3. (**f**) Model depicting CHOP-dependent regulation of miR-216b and CHOP-dependent apoptosis. Data are mean±s.d. of three independent experiments. Statistical analysis was analysed by Student's *t*-test (*P<0.05).

To investigate whether the effect of miR-216b is c-Jun-dependent, c-Jun was suppressed using three independent shRNAs targeting distinct regions of c-Jun. c-Jun loss triggered a significant increase in cleaved poly (ADP-ribose) polymerase (PARP) that correlated with the degree of c-Jun suppression (Fig. 7a). We compared the impact of overexpression of sh-c-Jun versus overexpression of miR-216b by serial increases in viral supernatants encoding either sh-c-Jun or miR-216b. This experiment revealed that while the artificial sh-c-Jun was significantly more potent in reducing c-Jun than miR-216b, the degree of cleaved caspase 3 triggered by either correlated with the degree of c-Jun knockdown (Fig. 7b). If loss of c-Jun triggers apoptosis, overexpression of c-Jun should over-ride ER stress-dependent and miR-216b-dependent apoptosis as assessed by caspase 3 cleavage. U2OS cells were infected with retrovirus coding c-Jun, lacking a 3′UTR, followed by TG treatment. Enforced c-Jun expression in U20S cells rescued cells from cell death induced by miR-216b expression and TG treatment (Fig. 7c,d). Finally, we reasoned that if miR-216b mediates CHOP-dependent apoptosis, enforced expression of miR-216b should sensitize CHOP^−/−^ MEFs to UPR-dependent cell killing. Consistently, enforced miR-216b expression resulted in increased cleaved caspase 3 in CHOP^−/−^ MEFs (Fig. 7e).

## Discussion

Activation of the UPR under varying stress conditions triggers an alteration in cellular protein content through direct modulation of gene expression, micro-RNA biosynthesis and protein translation rates; this in turn impacts cell division and ultimately enforces cell fate determination^57^. The final outcome, cell adaptation or cell death, is influenced by both stress intensity and duration. Importantly, how stress intensity is sensed by the UPR transducers, PERK/Ire1/ATF6, and transmitted into a fate decision remains to be elucidated. The complexity of this is increased by genetic evidence revealing that both genetic and pharmacologic inhibition of anyone branch can either sensitize cells to apoptosis or delay onset of apoptosis^33^,^42^,^58^. Some of these paradoxical outcomes can be resolved when one considers either the type of stress or the cell/tissue type being stressed. However, such context still requires that downstream effectors of the primary transducers be selectively activated to achieve specific fates. The elucidation of how effectors interpret signals and regulate cell fate requires a detailed understanding of both pro-survival and pro-apoptotic signalling.

Accumulating evidence supports a role for differential regulation of micro-RNAs by the UPR in the regulation cellular fate decisions under conditions of stress. Indeed, all three UPR transducers have been associated with micro-RNA regulation^24^,^25^,^26^,^27^. Thus far, Ire1 activity has been generally associated with inhibition of micro-RNA accumulation through its RNase function. Targeted micro-RNAs include miRs-17, 34a, 96 and 125b (ref. 25). In contrast, PERK-dependent activities appear to be associated with both micro-RNA induction, miR-211/204, miR-708 (ref. 23) and suppression miR-106-25 (ref. 24). It is of interest that all of the PERK-dependent micro-RNAs have been linked to the pro-survival activities of PERK. In the work described here, miR-216b is demonstrated to accumulate via the coordinated activities of both PERK and Ire1. Strikingly, in contrast to other PERK-regulated micro-RNAs, miR-216b contributes to UPR-associated apoptosis through regulation of a newly identified target, c-Jun. Although c-Jun, a component of the AP-1 transcription factor^54^, has not previously been demonstrated to contribute to gene expression patterns associated with ER stress, activation of Jun kinase pathway (JNK), was previously linked with Ire1-dependent activation of apoptotic signalling kinase-1 (refs 59, 60). Accordingly, activation of JNK is associated with increased apoptosis, a consequence of JNK-dependent inactivation of Bcl-2 (refs 61, 62). Induction of miR-216b is not associated with alterations in JNK signalling; however, it also contributes to increased apoptosis through downregulation of c-Jun.

miR-216b accumulation peaks several hours into the stress response, suggesting it functions as cells commit to either survival or death. This conclusion is supported by a number of experiments. First, miR-216b expression is directly dependent upon CHOP activity, another pro-apoptotic factor. Second, reduction in miR-216b activity increases cell resistance to UPR-dependent cell death. Third, overexpression of a miR-216b increases apoptosis. Finally, we demonstrate that modulation of c-Jun, a bona fide target of miR-216b during ER stress, also has the expected outcome on cell fate during an ER stress response including loss increasing cell death during ER stress.

One of the more vexing issues associated with UPR-triggered apoptosis has been the CHOP transcription factor. Genetic evidence from both cell and animal models has revealed CHOP to be a pro-apoptotic transcription factor during cellular stress, whether ER stress or DNA damage^62^,^63^. Several mechanisms have been suggested to mediate the pro-apoptotic activity of CHOP. These include suppression of anti-apoptotic Bcl-2 expression^64^, inducing pro-apoptotic Bim and DR5 (ref. 64), increasing translation and thereby compromising cellular bio-energetics and redox homeostasis^65^. Critically, many of these activities are not only dependent upon PERK-dependent activation of CHOP expression, but also depend upon ASK1-dependent activation of p38^MAPK^, which phosphorylates and induces CHOP^66^ demonstrating key points of Ire1 and PERK convergence on the establishment of an apoptotic fate under high stress conditions. Our data, which demonstrates CHOP-dependent induction of miR-216b, reveal yet another point of pro-apoptotic convergence wherein miR-216b antagonizes c-Jun accumulation and thereby promotes cell death.

The collective data suggest that CHOP-dependent apoptosis is unlikely to reflect a single functional target. Rather, as with many transcription factors, it is the additive function of several downstream targets that facilitate the establishment of cell fate. In this model, CHOP serves as a focal point downstream of PERK and Ire1 action. CHOP-dependent transcription of targets such as Bcl-2, BIM and miR-216b, the latter of which then targets an increasing number of transcripts, c-Jun as one example, thereby amplifying the pro-apoptotic activity of CHOP (Fig. 7f). As the action of miR-216b is delayed, by virtue of the time needed for its induction and targeting its substrates, this latency likely provides a needed opportunity for cells to repair and adapt if possible prior to initiating apoptosis.

## Methods

### Cell culture

NIH3T3 mouse embryonic fibroblasts (American Type Culture Collection (ATCC)) and murine embryonic fibroblasts 293T human embryonic kidney cells (ATCC) were maintained in DMEM (Mediatech) with 10% heat-inactivated fetal bovine serum (BenchMark). In addition, MEF cells were supplemented with 10% fetal calf serum, 2 mM L-glutamine, 55 μM β-mercaptoethanol and MEM nonessential amino acid mix (containing alanine, aspartate, asparagine, glutamate, glycine, proline and serine), all from Invitrogen. Human U2OS human cervical adenocarcinoma cells (ATCC) were maintained in McCoy's 5A (Mediatech) with 10% heat-inactivated fetal bovine serum, pen/strep.

### Plasmids and chemicals

Chop (Plasmid #21914) and Xbp1s (pCMV5-Flag-XBP1s) expression vectors were purchased from Addgene. Plasmids encoding sh-cJun (TRCN0000039590 and TRCN0000039589 and TRCN0000039592) were purchased from Daharmacon. PERK inhibitor is purchased from Selleckchem (GSK-2606414).

### MicroRNA microarray and qPCR

NIH 3T3 cells were challenged with 2 μg ml^−1^ Tm for 2, 5 or 10 h. Total RNA was purified using miRNeasy Mini Kit (Qiagen). Microarray was carried out using GeneChip miRNA Arrays (Affymetrix) and results were analysed using GENESPRING software. analysis of variance and *t*-tests were used to calculate fold change and *P*-values. Spotfire (Somerville) software was used to generate heat maps. For qPCR, total RNA was purified after appropriate treatments using Ambion microRNA purification kit mirVANA (Ambion) as per the manufacturer's instructions. All microarray data were deposited and can be accessed via GSE79455. RNA was reverse transcribed using specific primers with microRNA reverse transcription kit (Applied Biosystems). qPCR was performed on an Applied Biosystems 7900 qRT-PCR machine (Applied Biosystems). MicroRNA was reverse transcribed using TaqMan MicroRNA Reverse transcription kit with specific Taqman RT primers for mmu-miR-216b and miR-202 (Cat. # 4427975, Applied Biosystems) as per the manufacturer's protocol. qPCR was performed with mmu-miR-216b and miR-202 Taqman primers using no AmpErase UNG Master mix (Applied Biosystems). Primers for mouse c-Jun: forward 5′-TGGGCACATCACCACTACAC-3′; reverse 5′-TCTGGCTATGCAGTTCAGCC-3′. Primers for human c-Jun: forward 5'-GAGCTGGAGCGCCTGATAAT-3'; reverse 5'-CCCTCCTGCTCATCTGTCAC-3'. Primers for human COL1A1: forward 5′-AGTGGTTTGGATGGTGCCAA-3′; reverse 5′-GCACCATCATTTCCACGAGC-3′. Primers for human DNMT1: forward 5′-CATGAGTGCATTGGTGGCTG-3′; reverse CTTCCACGCAGGAGCAGAC. Primers for human MTS: forward 5′-GTACTCGGGCAAAGAGGGTG-3′; reverse 5′-TTGTCCCTGTTGCTGTCCAA-3′. Primers for human TMP1: forward 5′-GGTCCTTTCCGACAAGCTGA-3′; reverse: 5′-TGAGCGTACAGCTCGTCTTC-3′.

### Luciferase assays

Oligonucleotides containing three copies of the miR-216b seed sequences were cloned into the 3′UTR of the firefly luciferase gene in pRLTK. The 1,000 bp upstream of mmu-miR-216b promoter was amplified by PCR and cloned into pGL4.11 using primers: forward 5′-GATCTCGAGGTTCCTTACTGGCTTGCT-3′and reverse 5′-CTGAAGCTTTTGTCAGACTGAAGAATC5-3′. The mutant mmu-miR-216b promoter mutant was generated using the QuikChange kit (Stratagene). Mutagenic primers used were as follows: forward 5'-CAGCTCTCTCACCTATATTGACCACACTGCAAAC-3'; reverse 5'-GTTTGCAGTGTGGTCAATATAGGTGAGAGAGCTG-3'. The 3X AP-1 reporter contains three canonical AP-1-binding sites (TGACTCA) upstream of a minimal promoter fragment containing a TATA box in the luciferase reporter site luciferase^67^. For luciferase assays, cells were transfected with the constructs and pRLTK (Addgene) vector, to normalize for transfection efficiency and protein synthesis, with Lipofectamine plus method and treated with TG (500 nM) for the time as indicated.

### Western blot analysis and ChIP

Cells were harvested by gently scraping and resuspended in EBC buffer (50 mM Tris-HCl, pH 7.5, 100 mM NaCl, 0.5% Nonidet P-40) supplemented with protease inhibitors. The cells were disrupted by sonication and then cleared by microcentrifugation at 10,000*g* for 10 min. Proteins were resolved by SDS–polyacrylamide gel electrophoresis, transferred onto nitrocellulose membrane and analysed by immunoblot. Antibodies used were as follows: PERK rabbit polyclonal antibody (Rockland, #100-401-962; 1:1,000 dilution), p-eIF2α mouse monoclonal antibody (Cell Signaling, #3597; 1:1,000 dilution), ATF4 antibody (Cell Signaling, #11815; 1:1,000 dilution), CHOP (Cell Signaling, #2895; 1:1,000 dilution), Ire1α (Cell Signaling, #3294; 1:1,000 dilution), c-Jun (Cell Signaling, #9165; 1:1,000 dilution), JunB (Cell Signaling, #3753; 1:1,000 dilution), actin (Sigma, #A2228; 1:20,000 dilution), lamin A (Abcam, ab26300; 1:1,000 dilution), cleaved caspase 3 (Cell Signaling, #9661; 1:1,000 dilution), PARP (Sigma, #P7605; 1:1,000 dilution). Antibody binding was visualized by enhanced chemiluminescence (Perkin Elmer). Uncropped scans of western blots are provided in Supplementary Fig. 6.

Chromatin was prepared using the truChIP Low Cell Chromatin Shearing Kit (Covaris) and sheared into 200–700 bp fragments using a Covaris S2 instrument (duty cycle, 2%; intensity, 3; 200 cycles per burst; 6 min). Immunoprecipitation was performed with IgG, ATF4 (Cell Signaling), or CHOP (Cell Signaling) antiserum. Quantification of the precipitated DNA was determined with qPCR (Qiagen, QuantiTect SYBR Green Mastermix) and normalized with the input genomic DNA. Primers used for CHOP and ATF4 binding to mmu-miR-216b promoter in NIH3T3 cells were: forward, 5′-AGCTCCTAGTGCACTCTCCTT-3′; reverse, 5′-AATGCCAGGCGATCTGTGT-3′; For U2OS cells, primers for CHOP: forward: 5′-CAGTGGGTCTTATGGAGGCAG-3′, reverse 5′-GTCTACTTGCAGGCTGACATGA-3′; for ATF4, forward primer 1: 5′-TCTCCTTTCACTTGGGCACC-3′, reverse primer 1: 5′-TCTCCTTTCACTTGGGCACC-3′; forward perimer 2: 5′-GGGCCAATGGACATCTACCAA-3′, reverse 2: 5′-CCTCTGGTGCCCAAGTGAAA-3′; the primers for ATF4 binding to CHOP promoter, forward: 5′-CAGCTTCTGGGGGAGACAAG-3′, reverse: 5′-CAACGGCTATCAGCCTTGGT-3′.

### Mir-216b cleavage assay

*In vitro* cleavage reactions were performed by using γ-32PATP-labelled oligonucleotides^68^. Xbp-1 mini (5′-CUGAGUCCGCAGCACUCAG-3′) or miR-216b (5′-AAAUCUCUGCAGGCAAAUGUG-3′; Integrated DNA Technologies) was labelled with γ-32P-ATP using T4 polynucleotide kinase (New England Biolabs). The labelled Xbp-1 and miR-216b were purified with G-25 Sephadex mini column (Roche, #11273990001). One microgram recombinant Ire1 (aa 465–977) or Ire1 (aa 465–977)/GST (Sino Biological Inc.) was incubated with 1 pmol of 32P-γATP-labelled Xbp1-mini or miR-216b in cleavage buffer (20 mM HEPES, pH 7.5, 50 mM NaCl, 1 mM dithiothreitol, 1 mM ATP) at 37 °C for 2 h. RNA cleavage products were resolved on a urea-19% polyacrylamide gel electrophoresis without phenol/chloroform extraction and then exposed to Blue Basic Autorad Film for 20–40 min.

### Clonogenic survival assay

Clonogenic assays were performed in U2OS stable cells^69^. Briefly, cells were plated at 2 × 10^3^ cells per 60 mm plates. Twenty-four hours later, they were exposed to 400 nM TG or 2.5 μg ml^−1^ Tm for the indicated times, then returned to growth medium. Fresh media were replenshished every 2 days. Viable colonies were stained with 3 ml of 0.5% Giemsa (v/v) for 5–10 min at room temperature at 12 days. Colony counts were normalized to plating efficiency and represented as fraction surviving compared with control (dimethylsulphoxide).

### Retroviruses and stable U2OS cells

293T cells were transfected with PMDL, VSVG, REV and pEZX containing miR-216b mimic (MmiR-3190-MR03, GeneCopoeia) or anti-miR-216b (miR-AN0324-AM03, GeneCopoeia) or pEZX empty vector (miR-0001-MR03, GeneCopoeia) as control using Lipofectamine Plus (Invitrogene). Viral supernatants were harvested 48 h after transfection, and were used to infect U2OS cells in the presence of 10 μg ml^−1^ polybrene. Retroviruses coding c-Jun or vector control were produced by co-transfection of ecotropic helper retrovirus plasmid and pBabe vector into 293T cells^70^. Selection to create stably A-miR-216b overexpressing cells was conducted with puromycin at 2 μg ml^−1^, or for anti-with 0.2 μg ml^−1^ Hygromycin B.

## Additional information

**Accession codes:** All microarray data were deposited and can be accessed via GSE79455.

**How to cite this article:** Xu, Z. *et al*. miR-216b regulation of c-Jun mediates GADD153/CHOP-dependent apoptosis. *Nat. Commun.* 7:11422 doi: 10.1038/ncomms11422 (2016).

## Supplementary Material

Supplementary Information Supplementary Figures 1-6
