# Supplementary material for: miR-216b regulation of c-Jun mediates GADD153/CHOP-dependent apoptosis
Source: Nat Commun. 2016 May 13;7:11422. doi: 10.1038/ncomms11422 (PMC4869177; doi:10.1038/ncomms11422)
Supplement: Supplementary Information — Supplementary Figures 1-6 [file ncomms11422-s1.pdf]

Supplementary Figure 1

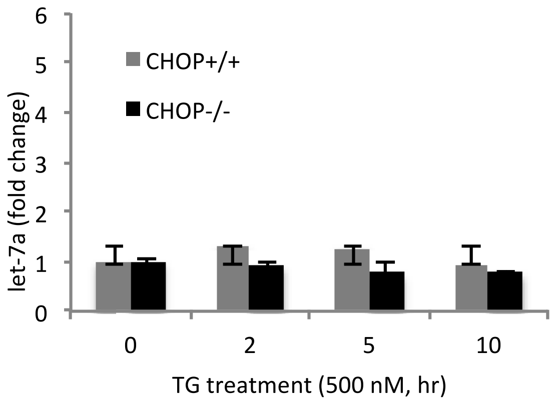

Supplementary Figure 1. qPCR analysis of let-7a in CHOP$^{+/+}$ and CHOP$^{-/-}$ MEFs treated with TG (500 nM) for indicated intervals. Data represent mean ± SD of three independent experiments. Statistical analysis were performed by Student's t-test.

Supplementary Figure 2

A

```
ccatgagcaataactatcagcattgcagagttgatattgtgatcatcaca
aattgtgctgttctcatatagtaaatactgcatattattttgttgcttta
aaaattagccgtagatgatgtgattaatctatgatgattagacagctact
aagtaataaaagacacatggggaaaatgaaggtgacaagtacagctccta
gtgcactctccttgtgcttttttagatatatctataagtttaaaaagctgt
gcagaaacagaaatgcaagttctagcacacagatcgcctggcattcaaga
aactccaggactgtgaagtttgcagtgtggtcaagcatcattataggtga
gagagctggtatttacctgttaccttagccttcagaagaacgtaatgcct
tttttcttgctcttgtgtttgaatcctatctatagtccttacttcctacc
taatgtcaatctttcataaatattttatgaaatgaataatatatgtttca
taaatataatttctgaaacataataattttagagtttagcaagactagaa
agaccattgtagagtgaaaaatggttttacatcttagatgtaattgcaca
TTGGCAGACTGGGAAATCTCTGCAGGCAAATGTGATGTCACTGAAGAAAC
CACACACTTACCTGTAGAGATTCTTCAGTCTGACAA
```

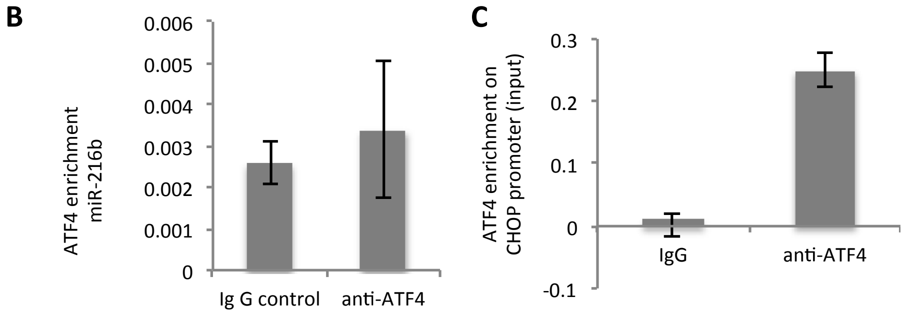

Supplementary Figure 2. (A) CHOP binding site -266 to -259 nucleotides upstream from TSS of mouse miR216b. **(B-C)** NIH3T3 cells were treated with TG (500 nM) for 5 hours, IgG or anti-ATF4 antibody was used for CHIP assay. The relative enrichment of ATF4 on the miR216b promoter (left panel) was confirmed by qPCR with specific primers. The success of precipitation of DNA was confirmed by the ATF4 enrichment on the CHOP promoter (right panel). Data represent mean ± SD of three independent experiments. Statistical analysis were performed by Student's t-test.

Supplementary Figure 3

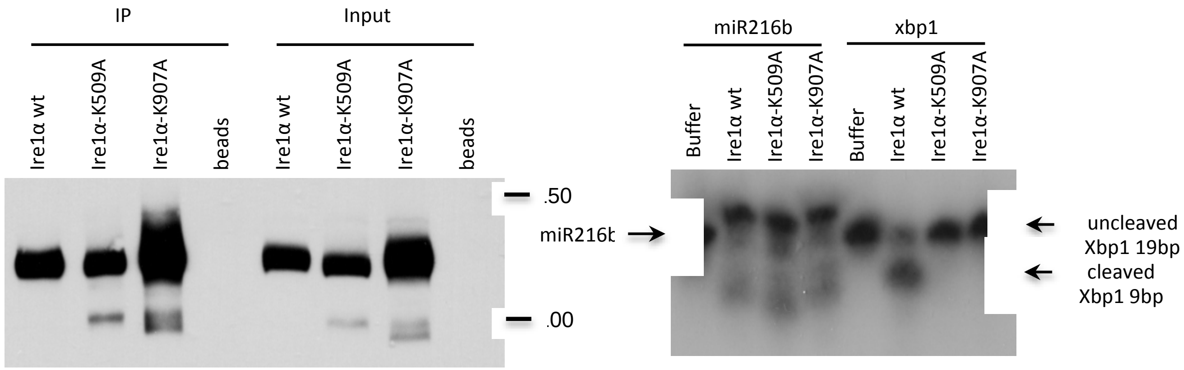

Supplementary Figure 3. (A) HA-tagged Ire1α wt, Ire1α-509A and Ire1α-907A were transfected into 293T cells and the proteins were immunoprecipitated anti-HA antibody. **(B)** γ-^{32}P ATP-labeled pre-miR216b and Xbp1 single stem-loop mini-substrate was incubated with immunoprecipitated proteins, and the cleaved products were resolved on a urea PAGE.

Supplementary Figure 4

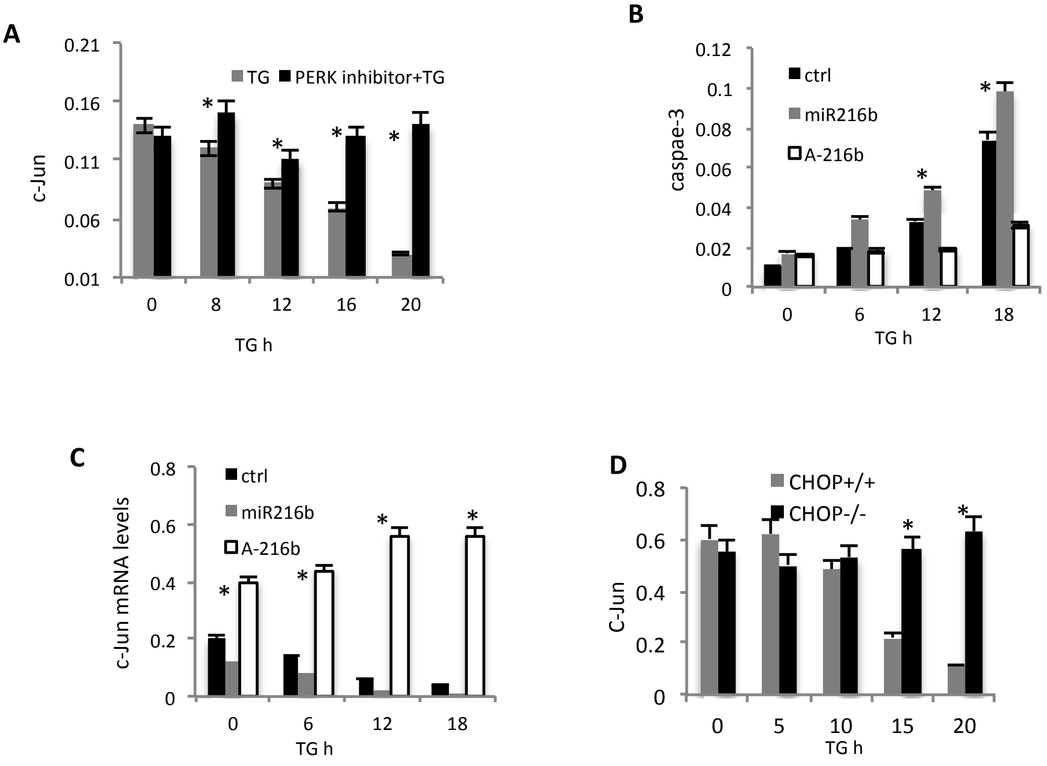

Supplementary Figure 4. MiR216b targets the c-Jun and sensitizes cells to ER stress

(A) Quantification of c-Jun in Fig 5C. **(B)** Quantification of c-Jun and caspase-3 Fig 5D (Normalized to actin). **(C)** c-Jun mRNA levels in cells from Fig 5D were quantified by q-PCR. **(D)** Quantification of c-Jun for figure 5J. Data represent mean ± SD of three independent experiments. Statistical significance were analyzed by Student's t-test. (*P<0.05)

Supplementary Figure 5

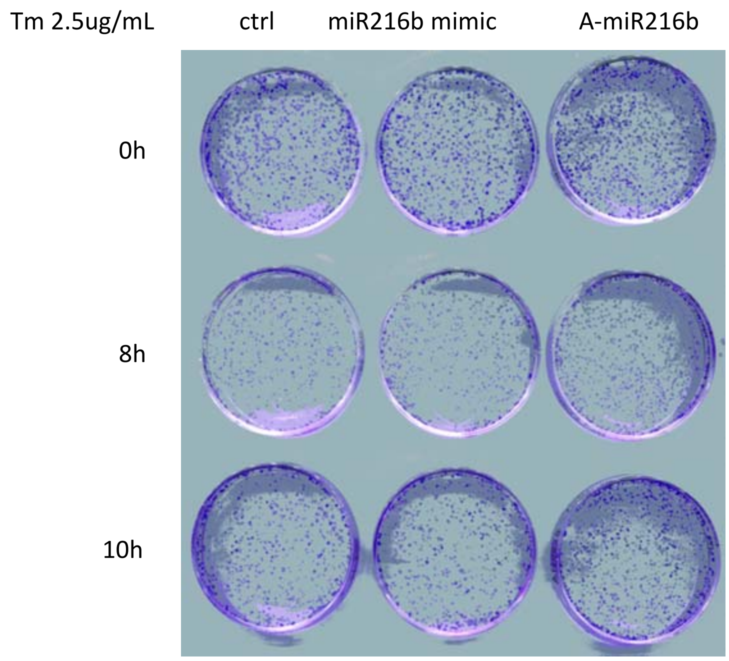

Supplementary Figure 5. (A) U2OS cells stably expressing control, miR216b mimic or A-miR216b were plated at 2X10^3 cells/60 mm dish, treated with Tm for 0, 8 or 10 hours, and then returned to growth medium for 12 days. Plates were assessed for colony formation by staining with Giemasa staining.

Supplementary Figure 6

Figure 2A

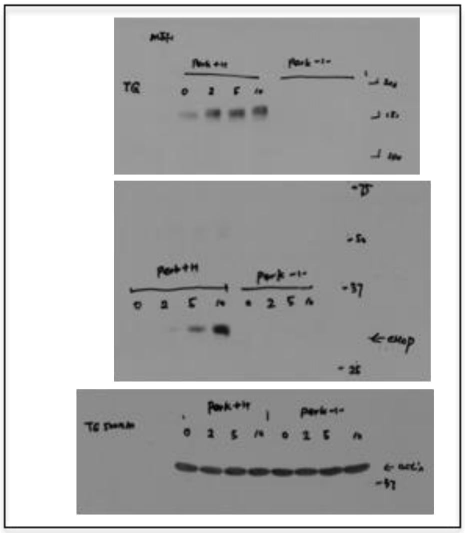

Figure 2B

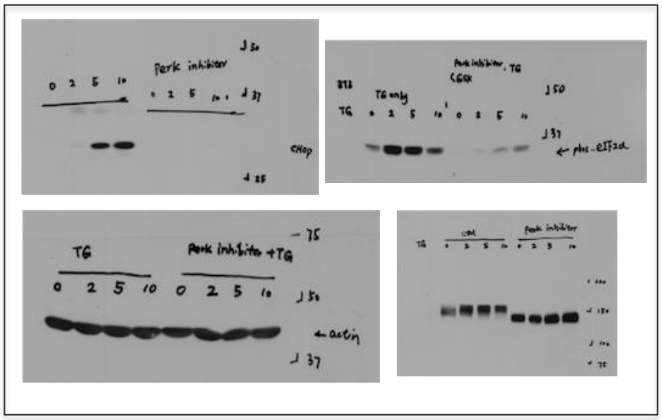

Figure 2D

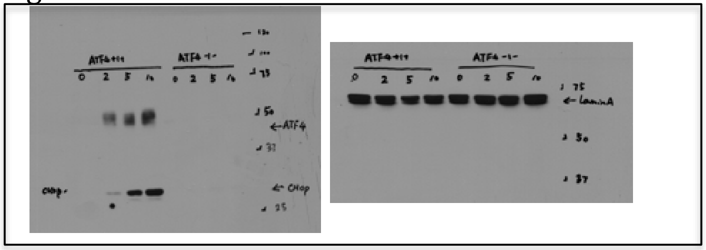

Figure 2C

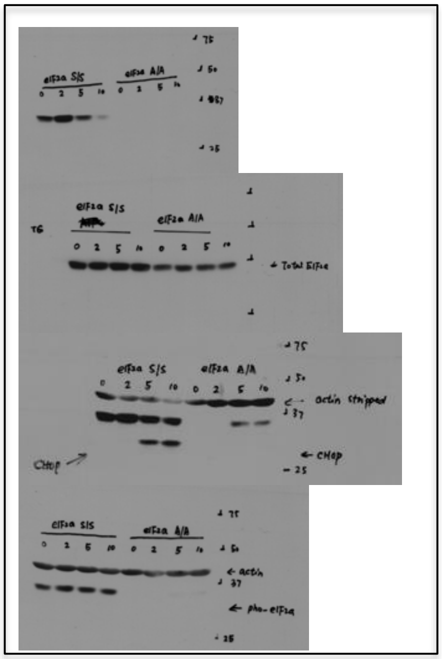

Figure 2E

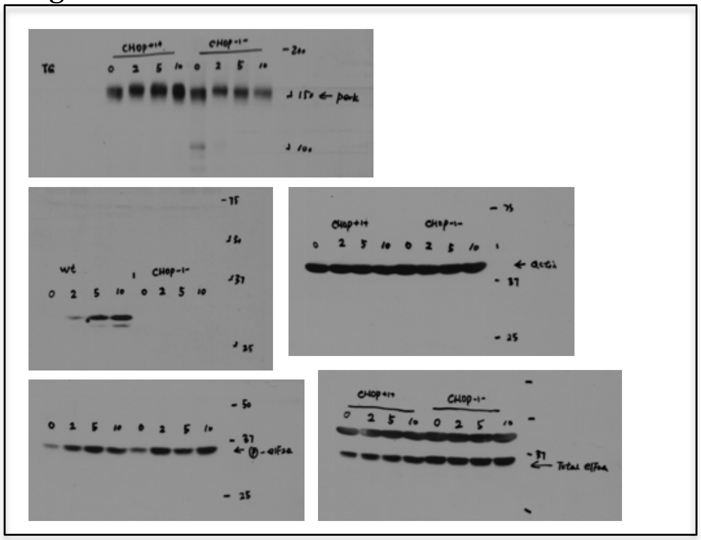

Figure 2F

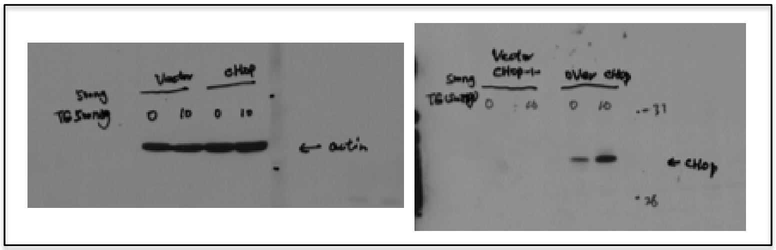

Supplementary Figure 6A
Full images of immunoblots in Figure 2.

Figure 4B

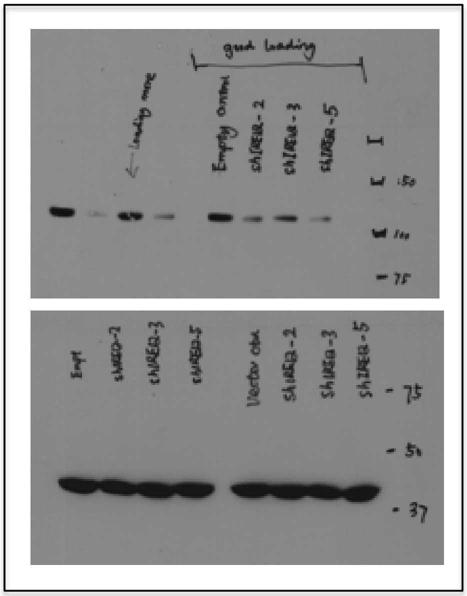

Figure 4D

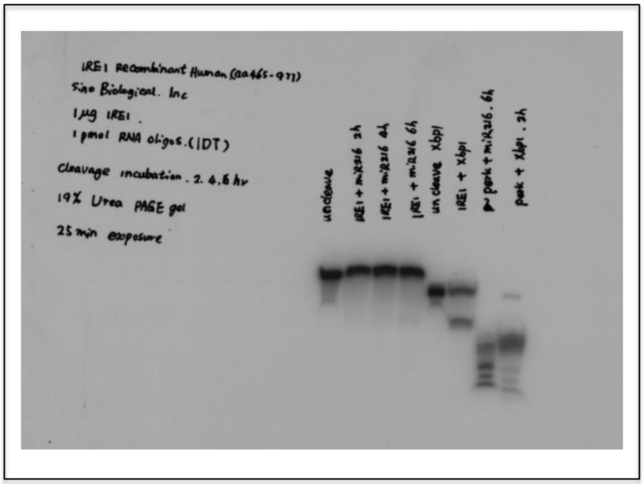

Figure 4E

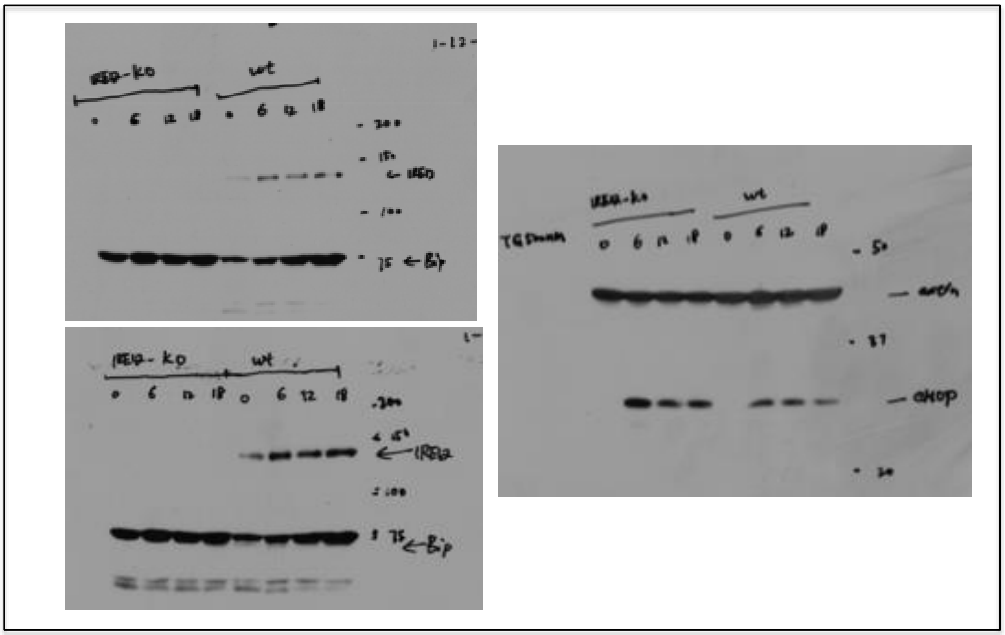

Supplementary Figure 6B Full images of immunoblots in Figure 4.

Figure 5C

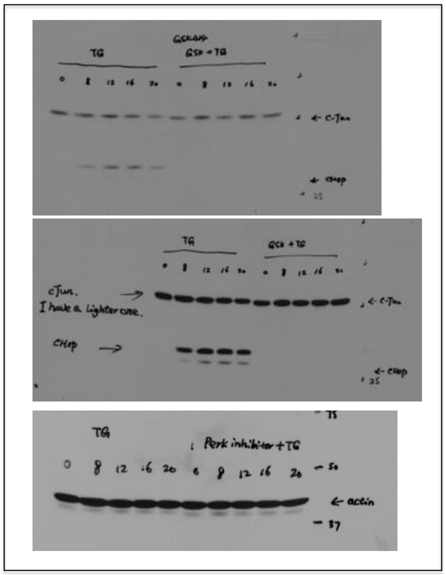

Figure 5D

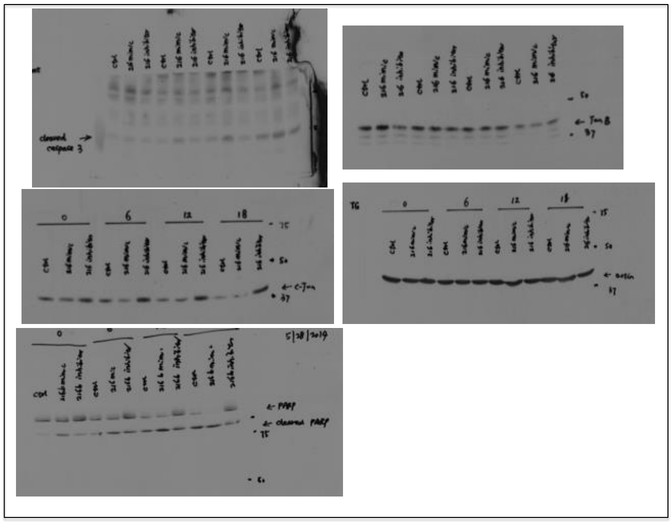

Figure 5F

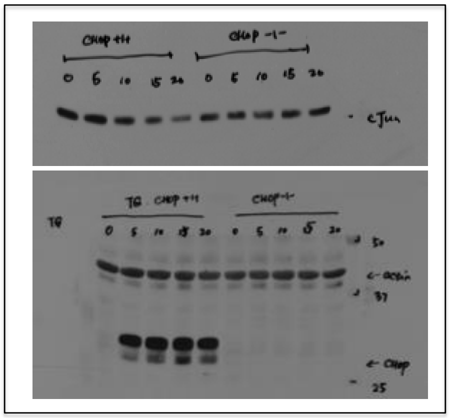

Figure 7B

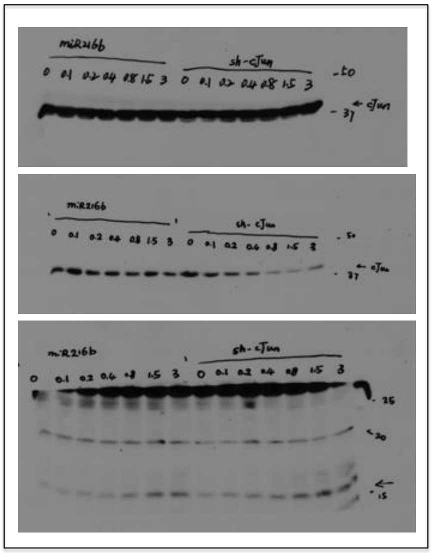

Figure 7E

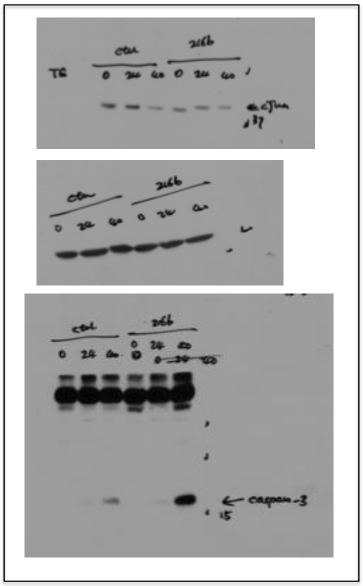

Supplementary Figure 6C Full images of immunoblots in Figure 5 and Figure 7.
